# Extracting cluster distributions from mass spectra: IsotopeFit

**DOI:** 10.1016/j.ijms.2015.01.004

**Published:** 2015-03-15

**Authors:** Stefan Ralser, Johannes Postler, Martina Harnisch, Andrew M. Ellis, Paul Scheier

**Affiliations:** a Institut für Ionenphysik und Angewandte Physik, Universität Innsbruck, Technikerstr. 25/3, A-6020 Innsbruck, Austria; b Department of Chemistry, University of Leicester, University Road, Leicester LE1 7RH, United Kingdom

**Keywords:** Cluster ions, Analysis software, Isotope patterns, Cluster size distributions

## Abstract

The availability of high resolution mass spectrometry in the study of atomic and molecular clusters opens up challenges for the interpretation of the data. In complex systems each resolved mass peak may contain contributions from multiple species because of the isotope structure of constituent elements and because a multitude of different types of clusters with different compositions are present. A computational procedure which can help to identify a specific cluster from this complex dataset and quantify its relative abundance would be extremely helpful to many who work in this field. Here some new software designed for this purpose, known as IsotopeFit, is described.

## Highlights

• Software for the evaluation of charged clusters is presented.
• Simple and complete extraction of cluster size distributions of all possible cluster series.
• Analysis of overlapping isotopic patterns in complex mass spectra.
• Freely available under a BSD license.

## Graphical abstract

**Figure:**
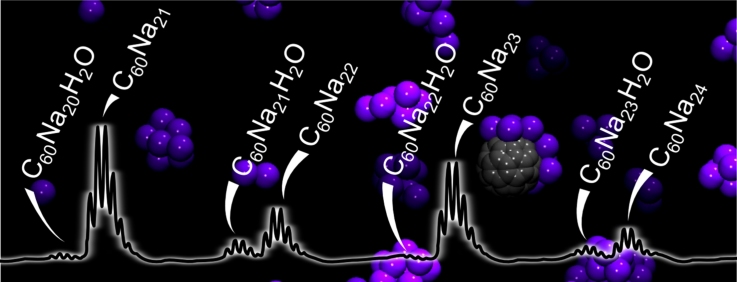

## 1 Introduction

In cluster physics it is often important to determine the relative abundance of certain cluster configurations from mass spectra, since information about the stability of clusters can be derived from this value. Of particular interest are so-called magic numbers, which were first reported by Echt et al. [1]. Evaluation of such data can be particularly complicated in the case of atoms with rather complex isotopic patterns, such as Kr [2] or other noble gases [3,4]. Other examples where establishing cluster sizes is important may be found in [5,6] and reviews by T.P. Martin [7,8]. In some of these studies clusters composed of more than one type of molecule have been investigated. A typical example for combined clusters of different molecules may be found in [9], where the adsorption of H_2_ molecules on a C_60_ surface was investigated by means of theory and experiment. Another area where there is high interest in determining the abundance of particular cluster sizes is the study of metal clusters [10,11].

Aside from the intentional study of clusters, mass spectrometry experiments using electrospray ionization [12] or matrix-assisted laser desorption ionization [13] sometimes yield clusters containing the original matrix material. These additional species are undesirable because they complicate the analysis of the mass spectra. An ability to extract the underlying contributions of specific analyte molecules from such mass spectra would be highly beneficial.

Ideally, what is required is some means of using the full range of isotope data to extract the underlying contributions of particular clusters to the observed mass spectral features. Here we describe new software that provides this capability. The computational process consists of several steps which are described in the following sections. The basic idea is to fit a computed spectrum with defined contributions from specific atomic or molecular monomers to the experimental data in order to retrieve the abundances of specific clusters or complexes. The software can correct for experimental artifacts such as background signal levels, the mass shift of the mass spectra, imperfect peak shapes and mass drift over time. In this communication we outline the algorithms used in the software package, which is known as IsotopeFit, and provide an example of its application.

## 2 Isotopic pattern calculation

An isotopic pattern *p*_*E*_(*m*) for an element *E* with *N*_*I*_ isotopes consists of *N*_*I*_ mass-abundance pairs and can be interpreted as a sum of weighted delta functions, where the abundances *a*_*i*_ have to fulfill the property $\sum_{i}^{N_{I}}a_{i}=1$. Exact masses *m*_*i*_ and relative abundances *a*_*i*_ for all isotopes of a certain element are well known and can be found in [14] and [15] respectively. The probability of observing a signal at mass *m* can be calculated using the following expression:

(1) $$p_{E}(m)=\sum_{i=1}^{N_{I}}a_{i}\delta (m-m_{i})$$

For a given *ν*-atom molecule M, containing the elements *E*_1_ to *E*_*ν*_, the resulting isotopic pattern *p*_*M*_(*m*) can be calculated via subsequent convolution of the isotopic patterns of its elements:

(2) $$p_{M}(m)=p_{E_{1}}*p_{E_{2}}*\cdots *p_{E_{\nu }}(m)$$

In the following we will use an artificial atom X to demonstrate the behavior of the different evaluation steps. We choose X to have the following isotope distribution:

**Table:** 

Mass	Abundance
1	0.2
2	0.8

**Fig. 1:**
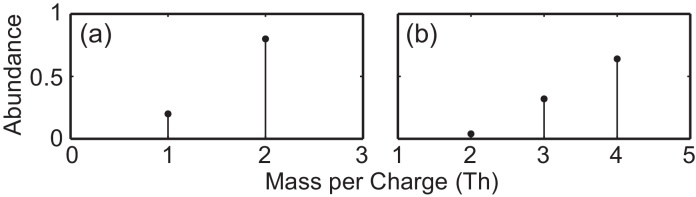
Isotopic pattern for an artificial atom X (a) and the calculated pattern for the molecule X_2_ (b).

Fig. 1 illustrates the distribution of the isotopes for the atom X and the calculated pattern for the dimer X_2_.

For a homonuclear molecule *E*_*ν*_, with *E* having *N* isotopes and *ν* being the number of atoms forming the molecule, the computation of its isotopic pattern scales in the order of *ν*^*N*^. To avoid computational overload, approximations are applied between every convolution step. The approximations used are:

1 Neglect of entries that have an abundance below a certain threshold *ϵ*_*a*_.
  $$a_{i}=0\;\mathrm{for}\;a_{i}<\epsilon_{a}$$
2 Combination of peaks whose mass separation is below a certain threshold *ϵ*_*m*_ (given by the resolution of the instrument) and therefore cannot be separated. The combined peak appears at the weighted center of mass of the two single peaks.
  $$a_{1}\delta (m-m_{1})+a_{2}\delta (m-m_{2})\approx (a_{1}+a_{2})\delta (m-(a_{1}m_{1}+a_{2}m_{2}))\;\mathrm{for}\;|m_{1}-m_{2}|<\epsilon_{m}$$

This method allows for efficient calculation of large (cluster) mass spectra containing many different atoms and/or molecules. For example, it is possible to calculate the isotopic patterns for every ion that is present in a mass spectrum of C_60_ multimers doped with other molecules. Typical mass spectra from our helium droplet experiments (e.g. [9]) can easily contain 5000 different complexes, where each consists of a combination of 100 or more atoms/molecules. Furthermore many mass spectra, especially for positively charged species, contain multiply charged ions with a charge of *z* · *e*. These appear at the *z*^th^ fraction of their nominal mass whereas the peak-width is not affected.

## 3 Data modeling

The signal *s*(*m*) of a measured mass spectrum for a molecule M appears as the convolution of the underlying isotopic pattern *p*(*m*) (scaled with some factor *A*) with a convolution core *κ*(*m*) (“peak shape”) that is dependent on the technique used and the experimental parameters.

(3) s(m)=A·p*κ(m)

**Fig. 2:**
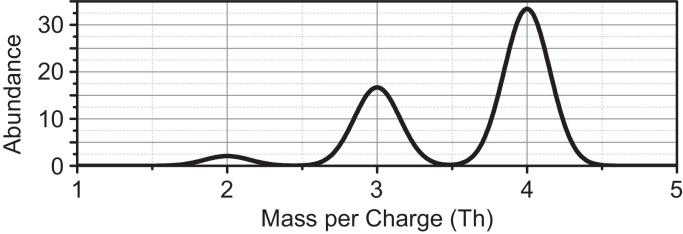
Constructed signal *s*(*m*) for the artificial molecule X_2_, convolved with a Gaussian core (*A* = 10, *R* = 10, *m*_0_ = 0).

As a simple example Fig. 2 shows a mass spectrum constructed for the X_2_ molecule.

The simplest and quite often also a reasonable function for the convolution core is a Gaussian peak shape. However, the IsotopeFit software has the ability to accept user-defined peak shapes.

The width of the Gaussian function *σ*, is inversely proportional to the instrument resolution *R* (in our case defined by the FWHM). The difference between the measured mass and the exact mass, which is known as the mass shift *m*_0_, is zero for an ideal instrument, but will be non-zero in every real spectrum due to experimental artifacts. Both parameters can vary over the whole mass range and have to be adapted to the experimental conditions via a calibration process (see the section on the fitting process).

For a certain resolution *R*, a molecular abundance *A* and an isotopic pattern *p*_*M*_(*m*), it is possible to construct the mass spectrum for the molecule M and compare it with the measured spectrum for this molecule. To model the data for the whole spectrum, one has to calculate a superposition of the spectra of every single molecule *M*_*i*_ that appears in the data:

(4) $$s_{\mathrm{calc}}(m)=\sum_{i=1}^{N}s_{i}(m)=\sum_{i=1}^{N}A_{i}\cdot p_{i}*\kappa (m)$$

*N* being the total number of molecules in the spectrum. This calculated spectrum *s*_*calc*_(*m*) constitutes the starting point for the fitting process to find the abundances *A*_*i*_ for every molecule involved.

## 4 Fitting process

In the previous section we have described how to model a mass spectrum by knowing the abundances *A*_*i*_ of the molecules involved (i.e. their sum-formulas), their isotopic patterns *p*(*m*), and the parameters *σ* and *m*_0_ of the convolution core. If we assume that we know the peak shape and the isotope pattern for all molecules involved, the problem transforms into a simple linear equation system where the parameters *A*_*i*_ can be found by minimizing the mean squared deviation between measured and computed signals.

Computationally, the solution for such a problem can be found by an optimization algorithm. Essentially a linear equation system with one equation for every signal-mass pair (*m*_*i*_|*s*_*i*_) of the measured spectrum is obtained where the abundances *A*_*i*_ are the unknowns. In the case of *k* points and *N* unknowns (molecules), a solution can always be found that minimizes the mean squared deviation between the calculated and measured spectrum, as long as we have more data points *k* than the number of unknowns *N*.

(5) $$s_{\mathrm{calc}}(m)=\left(\begin{array}{c} s(m_{1})\\ \vdots \\ s(m_{k}) \end{array}\right)=\left(\begin{array}{ccc} s_{1}(m_{1}) & \cdots & s_{N}(m_{1})\\ \vdots & \ddots & \vdots \\ s_{1}(m_{k}) & \cdots & s_{N}(m_{k}) \end{array}\right)\cdot \left(\begin{array}{c} A_{1}\\ \vdots \\ A_{N} \end{array}\right)=S\cdot \vec{A}$$

The optimization ∑_*i*_(*s*_*calc*,*i*_ − *s*_*exp*,*i*_)^2^ → min for the molecular abundances *A*_*i*_ is performed in IsotopeFit using the MATLAB^®^ function “lsqnonneg”, which implements mean squared deviation minimization for linear systems and automatically adds the constraining equations for non-negative optimization parameters.

### 4.1 Total number of counts per molecule

Most of the time we are not interested in the area *A* under a molecular pattern (*A* = ∑_*i*_*s*_*i*_Δ*m*_*i*_ will vary for different settings of the instrument), but rather want to know the total number of molecules A^*^ that contribute.

(6) $$A^{*}=\sum_{i}s_{i}\approx A\cdot \frac{1}{\Delta m}\;\mathrm{for}\;\Delta m=\Delta m_{i}=\mathrm{const}$$

IsotopeFit automatically applies this correction.

### 4.2 Error estimation

To estimate the goodness of the fitting parameters and to calculate the confidence intervals for the areas *A*_*i*_ in which the real values can be expected to be found, we need to know the covariance matrix for the area vector $\vec{A}$. It can be shown that this matrix can be estimated by calculating

(7) $$\mathrm{cov}(\vec{A})={\left(S^{T}S\right)}^{-1}\cdot \frac{1}{k-N}\sum_{i=1}^{k}{∥s_{\exp,i}-s_{\mathrm{calc},i}∥}^{2}$$

A 95% (two-sided) confidence interval is then found by taking the square root of the diagonal elements multiplied by the 0.975 quantile of Student's *t*-distribution with *k* − *N* degrees of freedom. The factor 1.96 is a good approximation for the 95% confidence level of a normal or Poisson distribution with a large number of degrees of freedom.

(8) $$\Delta \vec{A}=1.96\cdot \sqrt{\mathrm{diag}(\mathrm{cov}(\vec{A}))}$$

See [16] for further details.

## 5 Mass and resolution calibration

As shown before, we can find the abundances *A*_*i*_ in a single calculation step as long as we know the parameters for the convolution core. In general, these parameters are different for different masses (e.g. the resolution at high masses will be different than at low masses) and so one has to find a calibration curve in order to perform the fitting process for the abundances *A*_*i*_. A good method to find these parameters is to choose some “calibration-molecules” that have a high signal in the spectrum and can easily be identified. For these molecules we try to find the parameters for the convolution core *κ*(*σ*, *m*_0_) by performing a nonlinear fitting process. There are many different implementations of such algorithms in MATLAB^®^; we use the function fminsearch, which implements a simplex algorithm to minimize a test function with user-defined parameters [17]. As seen in the previous section, the abundances can be calculated in one step so the simplex algorithm has to search for only two parameters, namely the width *σ* and the mass-shift *m*_0_. To summarize, we adapt the following recipe:

1. Inspect the measured spectrum and find a molecule with a decent signal. Identify all molecules that are present in the chosen mass-range.
2. Calculate the convolution core *κ*(*σ*, *m*_0_) with a certain resolution and a mass offset. At the beginning, use a reasonable guess for the values of these parameters.
3. With this core, find the optimal abundances *A*_*i*_, as described in the section on the fitting process.
4. Calculate the mean squared deviation for the given data.
5. Proceed with variation of the convolution core parameters *σ* and *m*_0_ (simplex algorithm) and find the convolution core which best fits to the data by repeating steps 2 to 4.
6. Perform this optimization for different molecules in the spectrum to find the calibration curves for *σ*(*m*) and *m*_0_(*m*) over the whole mass range.

## 6 Convolution core modeling

To achieve the best data evaluation, it is important to know the exact shape of the mass peaks. In this section we present a way to find a model for the convolution core using the experimental data.

The starting point is an approximate knowledge about the abundances *A*_*i*_ of all molecules in a certain mass range. The chosen range should contain several peaks with a good signal-to-noise ratio. The abundances *A*_*i*_ can be estimated via the fitting process described above, using a Gaussian core (or any other reasonable peak shape). With these estimated abundances, the distribution *p*_*sum*_(*m*) of all peaks in this mass range can be calculated. Note that the guessed areas *A*_*i*_ are included in this distribution:

(9) $$p_{\mathrm{sum}}(m)=\sum_{i=1}^{N}A_{i}p_{i}(m)$$

The convolution core which we want to derive is the function that *p*_*sum*_ has to be convolved with in order to obtain the measured data. This convolution is a point-wise multiplication in the mass-frequency subspace (*ω* = 2*π*/*m*):(10)

In this subspace, the convolution core can be derived via a point-wise division where a Fourier back-transformation of *K*(*ω*) gives the convolution core *κ*(*m*).

Depending on the quality of the data the derived core will be more or less noisy. To remove this noise and to provide a peak-shape model that is easy to process, a cubic spline with a chosen number of points is fitted to the raw core. This spline can then be normalized in width and area in order to use it with the software.

## 7 Background correction

**Fig. 3:**
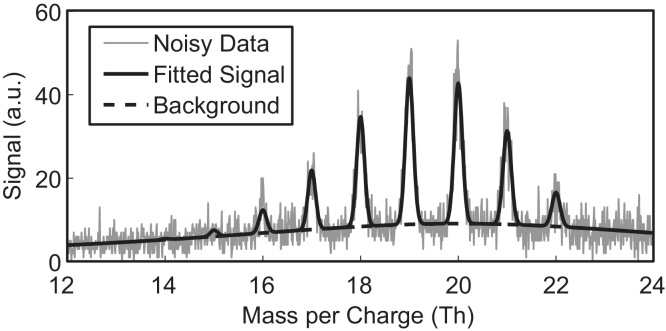
Simulated data for a mixture of the molecules X_10_ and X_11_ with an abundance of $A_{X_{10}}=10$ and $A_{X_{11}}=20$. The convolution core parameters were *R* = 100 and *m*_0_ = 0. The background level varies over the mass range and was modelled with a Cauchy distribution.

Every mass spectrum has a certain level of background counts (dark counts, misguided electrons, electronic noise, etc.) that needs to be corrected for in order to be able to evaluate the data properly. Fig. 3 shows a typical (simulated) mass spectrum with added noise and background level for the artificial clusters X_10_ and X_11_.

Our algorithm to correct the background level divides the mass spectrum into a specific amount of smaller sub ranges, which can be varied by the user. For each of these sub ranges, a certain background level to be considered noise is given as a parameter by the user in terms of number of data points (in percent). The algorithm calculates the mean signal value of those data points considered noise and fits a piecewise cubic hermite interpolation polynomial through the data points. As opposed to a normal spline, the cubic hermite interpolation has the additional restriction of aiming to keep the monotonicity of the data, as described in detail in [18].

Before the actual fitting process, this background level has to be subtracted from the data.

## 8 Drift correction

Mass spectra can show a so-called “mass drift” under specific circumstances. This refers to a change in the mass scale over time, usually due to a temperature change of the instrument. When such a shift is averaged over a long-time measurement it can cause a loss in resolution that is easily correctable if the time evolution of the spectrum is recorded. This is usually the case for TOF instruments.

**Fig. 4:**
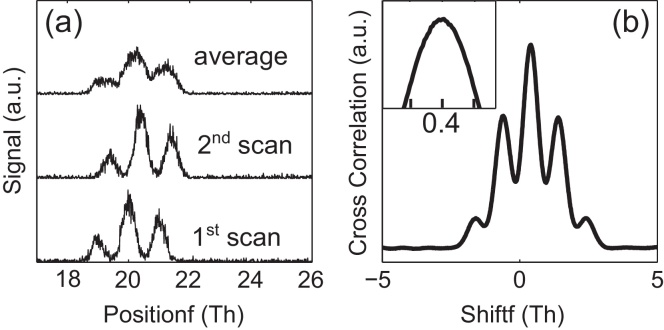
(a) Simulated noisy data with a mass drift of 0.4 Th between the lowest two scans. The upper plot shows the averaged spectrum with a loss of resolution due to drift effects. The calculated cross correlation between the two scans is shown in (b).

To correct for this drift we take different averaged spectra at different times of the measurement and look at the cross correlation (along the whole mass range) in order to model the drift in time. Fig. 4 illustrates this process. Once the drift is known, we can calculate the corrected averaged spectrum with the nominal instrument resolution.

## 9 Example: data evaluation for C_60_ + Na complexes

### 9.1 Experimental data

In this section we show an example for a data evaluation achieved with IsotopeFit. The experiment was undertaken with a helium nano-droplet source with doping of the droplets by C_60_ and Na. This investigation was performed with a ToF-MS described in detail elsewhere [19]. The data shows cationic fullerene complexes decorated with sodium atoms up to a mass of 5000 amu. To avoid unnecessary diversion the physics of the experiment will not be discussed here. The time to evaluate this spectrum was roughly 2 h for the determination of the ions involved and the precise calibration of the spectrum. The calculation time for the fitting process is, depending on the amount of complexes included, 5 min up to several hours on a quadcore desktop computer. More cores are beneficial, as the matrix operations can be parallelized.

### 9.2 Evaluation

For the evaluation, a set of the following ideal isotopic patterns was generated:

• ${\left[{\left(C_{60}\right)}_{n}{\left(\text{Na}\right)}_{m}{\left(H_{2}O\right)}_{j}\right]}^{+}$ with 1 ≤ *n* ≤ 10, 0 ≤ *m* ≤ 40, 0 ≤ *j* ≤ 1
• ${\left(\mathrm{He}\right)}_{n}^{+}$ with 1 ≤ *n* ≤ 200
• ${\left(\mathrm{Na}\right)}_{n}^{+}$ with 1 ≤ *n* ≤ 100
• several doubly charged species (such as $C_{40}^{++}$)
• several contaminants and other contributions

In total, a set of 3430 different isotopic patterns was used in the evaluation process.

**Fig. 5:**
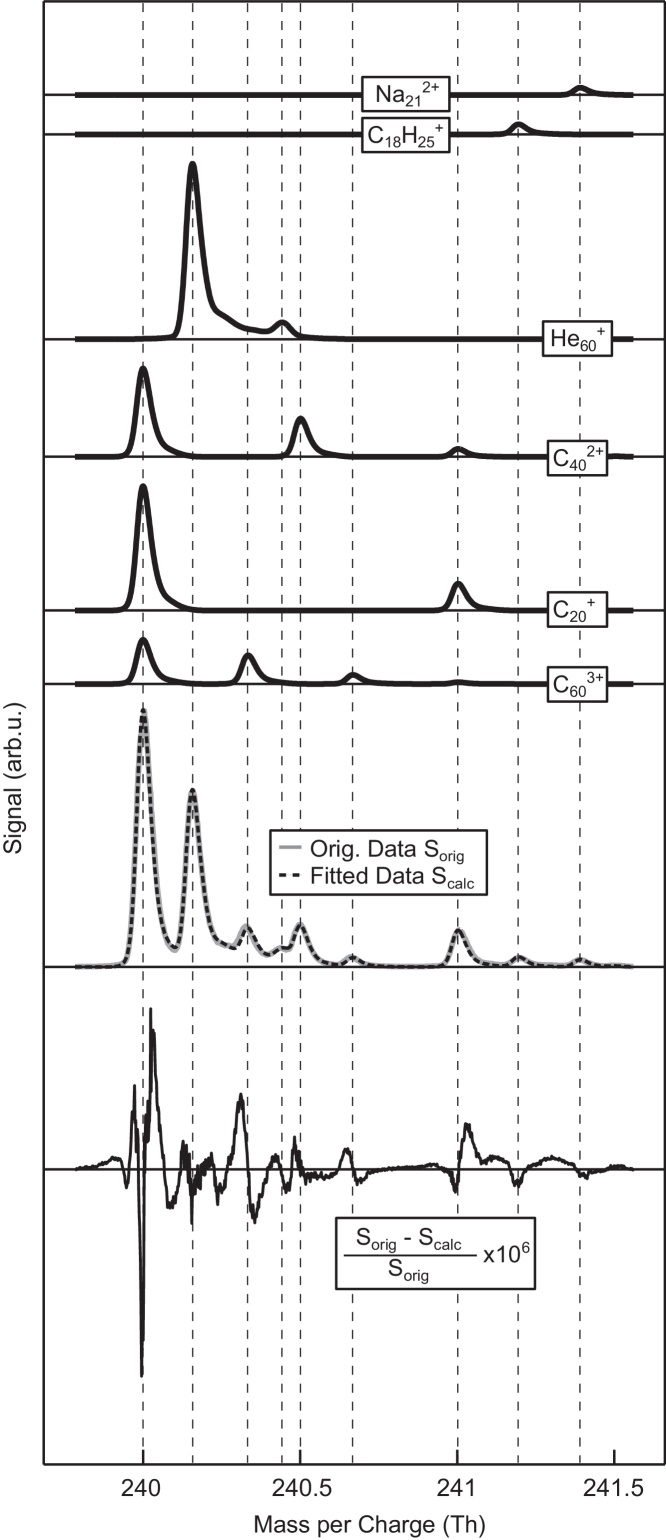
Comparison between the measured and the calculated data for a small section of the spectrum. The upper curves show the different contributions to the total mass spectrum. Also shown is the relative difference between measured and calculated data scaled by factor of 10^6^.

In order to achieve a satisfying evaluation, we used a custom convolution core derived with the technique described above. This core is different for helium-containing peaks as opposed to pure carbon peaks due to contributions from metastable helium cluster ions to the former [20]. Fig. 5 shows part of the spectrum, which contains contributions from six different ions (${\mathrm{Na}}_{21}^{2+}$, $C_{18}H_{25}^{+}$, ${\mathrm{He}}_{60}^{+}$, $C_{40}^{2+}$, $C_{20}^{+}$, $C_{60}^{3+}$). One can easily see the specific contributions of each ion involved in the fitting procedure resulting in the complete spectrum measured. The lower part of the plot shows the residual signal. Note that the residual signal is about a factor of 10^6^ smaller than the ion signal. For $C_{60}^{3+}$ the peaks from the two lightest and most abundant isotopologues are hidden among large contributions from other ions. Nevertheless the underlying contribution from $C_{60}^{3+}$ can be ascertained automatically within the fitting process employed within IsotopeFit. This means that IsotopeFit can readily provide relative abundances of specific clusters whereas a manual approach would be slow and fraught with complications from overlapping peaks.

**Fig. 6:**
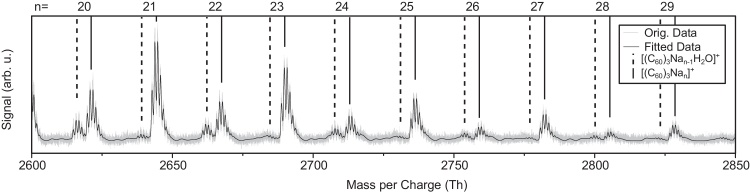
Part of the Na + C_60_ mass spectrum in the range of 2600–2850 thomson showing both the fitted and the measured data. The solid lines mark complexes of the form ${\left[{\left(C_{60}\right)}_{3}{\mathrm{Na}}_{n}\right]}^{+}$, whereas the dashed lines mark the same complexes with the addition of an H_2_O molecule.

Fig. 6 shows a wider section of the mass spectrum with the fitted spectrum as an overlay. The solid lines identify complexes of the form ${\left[{\left(C_{60}\right)}_{3}{\mathrm{Na}}_{n}\right]}^{+}$, whereas the dashed lines mark the same complexes with the addition of an H_2_O molecule. As can be seen the agreement between the simulated and experimental spectrum is excellent.

**Fig. 7:**
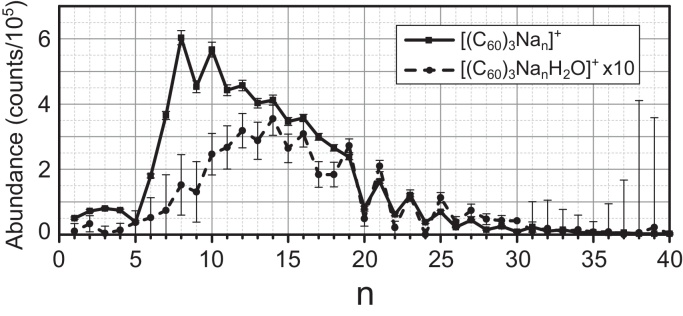
Abundance for complexes of the form ${\left[{\left(C_{60}\right)}_{3}{\mathrm{Na}}_{n}\right]}^{+}$ (solid line) and of the form ${\left[{\left(C_{60}\right)}_{3}{\mathrm{Na}}_{n}H_{2}O\right]}^{+}$ (dashed line) for different *n*. Note the different behavior of the two types of complexes and the odd-even-oscillations for *n* > 19.

As already outlined in the introduction, the main purpose of IsotopeFit is to generate reliable “cluster series”, i.e. lists of abundances for various complexes present. An example for such a cluster series is shown in Fig. 7, where a typical feature of such data, an odd-even-oscillation can easily be seen for *n* > 19. Also note the different behavior of complexes of the form ${\left[{\left(C_{60}\right)}_{3}{\mathrm{Na}}_{n}H_{2}O\right]}^{+}$ from the ${\left[{\left(C_{60}\right)}_{3}{\mathrm{Na}}_{n}\right]}^{+}$ series for *n* < 20.

## 10 Discussion and summary

In this work we describe new software that can be used for the evaluation and interpretation of complicated mass spectra and is specifically aimed at solving problems in cluster physics. By convolution of isotopic patterns spectra containing many different atoms/molecules can be calculated. The measured signal can be reproduced by scaling these isotopic patterns with the peak areas and convolving them with a convolution core (dependent on the shape of the mass peaks). The different steps necessary to perform this data modeling (i.e., background correction drift correction, mass and resolution calibration and the fitting process) have been explained in detail in this work. The software was tested on several different kinds of cluster mass spectra measured by the CLUSTOF experiment [19] at the University of Innsbruck.

**Fig. 8:**
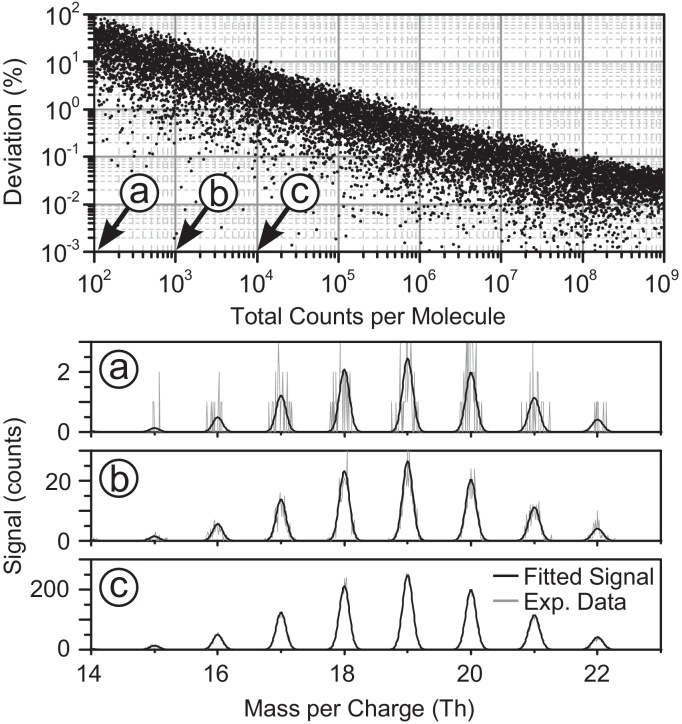
The upper panel shows 10,000 evaluations for a simulated spectrum with a 1:1 ratio of X_10_ and X_11_ clusters. Plotted is the normalized deviation between fitted abundance of X_10_ and its nominal abundance versus increasing signal quality. The panels a, b and c illustrate 3 different signal qualities. The positions of these simulations are marked in the upper panel.

The software provides reliable results for a minimum in the order of 5 × 10^3^ total counts per compound (see Fig. 8). For noisier spectra different evaluations of the same data lead to a spread of the fitted abundances which are reflected in error bars for the cluster series plots.

## 11 General notes

• **License:** The code is freely available under a BSD 3-clause license.
• **Language:** IsotopeFit was written in MATLAB^®^ and requires version 2013b.
• **Availability:** A git repository is available on Github^®^ under the following URL: https://github.com/nano-bio/IsotopeFit. Any contributions in the form of code or bug reports are highly welcome.
• **Platform:** IsotopeFit has been tested with Scientific Linux 6.5, Ubuntu 14.04 and Windows^®^ 7.
• **Status:** Currently IsotopeFit is in a state of functional preview and tailored to the needs of the authors. Further improvements and generalizations may be included in the future.
